# Pleomorphic lipoma lacking mature fat component in extensive myxoid stroma: a great diagnostic challenge

**DOI:** 10.1186/1746-1596-7-155

**Published:** 2012-11-13

**Authors:** Xu-Yong Lin, Yan Wang, Yang Liu, Ying Sun, Yuan Miao, Yong Zhang, Juan-Han Yu, En-Hua Wang

**Affiliations:** 1 Department of Pathology, the First Affiliated Hospital and College of Basic Medical Sciences, China Medical University, Shenyang, 110001, China; 2 Institute of pathology and pathophysiology, China Medical University, Shenyang, 110001, China; 3 Department of Outpatient, the First Affiliated Hospital, China Medical University, Shenyang, 110001, China

**Keywords:** Pleomorphic lipoma

## Abstract

Pleomorphic lipoma is a relatively uncommon entity, and is considered as a variant of spindle cell lipoma. Histologically, spindle cell lipoma/pleomorphic lipoma consists of varying quantity of mature fat, bland spindle cells and ropey collagen. In addition, pleomorphic lipoma is characterized by multinucleate giant cells, which possess the “floret-like” nuclei and marked pleomorphism. So, in contrast to spindle cell lipoma, pleomorphic lipoma is more easily misdiagnosed as a malignant tumor. Herein, we report a peculiar case of pleomorphic lipoma occurring in axilla with entirely devoid of mature fat in a 71-year-old male. The histopathological findings demonstrated the tumor was made up of bland spindle cells admixed with scattered “floret-like” cells and irregular ropey collagen in an extensive myxoid stroma. Immunostaining showed that the tumor was positive for the Vimentin, Bcl-2 and CD34, and was negative for S-100, desmin, CD68, and α–SMA. Although no fat component was found in the whole section, the tumor was still diagnosed as a pelomprphic lipoma. To our knowledge, this is the third reported case of pelomprphic lipoma which entirely lacked lipomatous component. Because of the existence of atypical multinucleate giant cells and lack of mature fat, this tumor may be easily misdiagnosed nonlipomatous lesions, such as myxoid fibrosarcoma, giant cell fibroblastoma.

So, it is necessary to pay careful attention to the histological spectrum of pleomorphic lipoma, including the tumor with devoid of fat, and it should be kept in mind that pelomprphic lipoma still can be diagnosed even if lacking lipomatous component.

**Virtual slides:**

The virtual slide(s) for this article can be found here: http://www.diagnosticpathology.diagnomx.eu/vs/1967123180611361

## Introduction

Pleomorphic lipoma was first described by Shmookler and Enzinger in 1981 [1], 6 years later than the first description of spindle cell lipoma by Enzinger and Harvey [2]. The two tumors display an overlapping histological feature [1-3], similar immunohistochemical [4,5], and cytogenetic features [6-8]. So, pleomorphic lipoma is considered as a variant of spindle cell lipoma.

Pleomorphic lipoma occurs predominantly in the subcutaneous tissue of the posterior neck, shoulder, and back [1]. Less frequently, it can occur in unusual locations including palm [9], tonsillar fossa [10], orbit [11], tongue [12], vulva [13] and oral cavity [14].

Microscopically, pleomorphic lipoma can show variable histological appearances. The majority of the tumors show varying proportion of mature fat, spindle cells and “floret-like” cells. Some tumors may predominantly consist of adipose tissue with scattered spindle cells or “floret-like” cells, others may predominantly consist of spindle cells and “floret-like” cells with a little adipose tissue. Very rarely, it may entirely lack mature adipocytes which can pose a great challenge, and be misdiagnosed easily.

So, to avoid the misdiagnosis, it is necessary to be familiar with the histological spectrum of this entity. Here, we report a case of pleomorphic lipoma arising in axilla with fat component absent and extensive myxoid change in a 71-year-old Chinese male. It should be differentiated from some lesions including myxoid fibrosarcoma, giant cell fibroblastoma, pleomorphic fibroma and giant cell angiofibroma.

## Case history

A 71-year-old male referred to our hospital with feeling of a painless swelling in the subcutaneous tissue of the right axilla. Physical examination revealed a 5-cm subcutaneous mass which felt firm and moved well. Ultrasonography revealed a low echo mass measuring in diameter 48 mm in the subcutaneous fat tissue of the right axilla, the mass was well circumscribed. Then the tumor was excised and underwent biopsy.

## Materials and methods

The submitted specimens were fixed with 10% neutral-buffered formalin and embedded in paraffin blocks. Tissue blocks were cut into 4-μm slides, deparaffinized in xylene, rehydrated with graded alcohols, and immunostained with the following antibodies: Vimentin, CD34, S-100, desmin, α–smooth muscle actin (α–SMA), Bcl-2, CD68 and Ki-67. Sections were stained with a streptavidin-peroxidase system (KIT-9720, Ultrasensitive TM S-P, MaiXin, China). The chromogen used was diaminobenzidine tetrahydrochloride substrate (DAB kit, MaiXin, China), slightly counterstained with hematoxylin, dehydrated and mounted.

## Results

**Figure 1:**
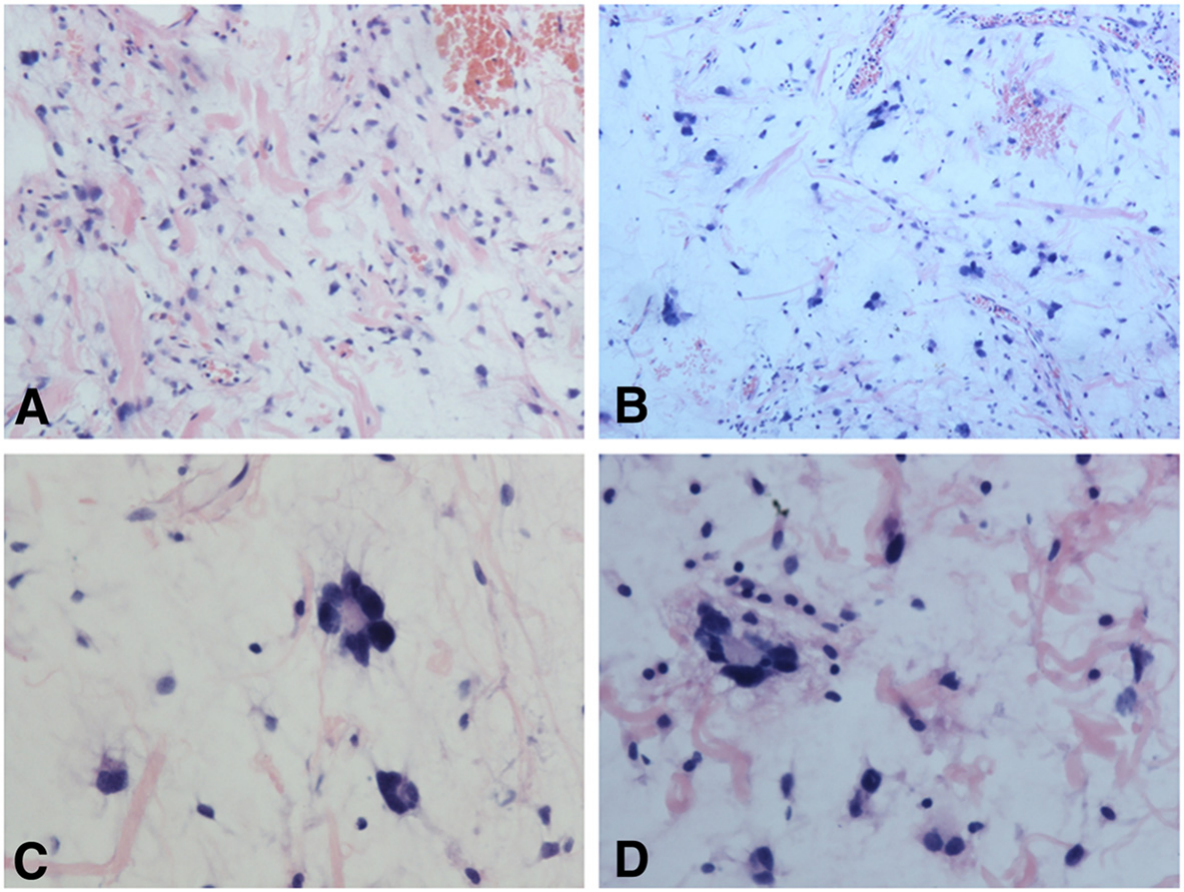
**A,The tumor predominantly consists of spindle cells admixed scattered multinucleated cells and abundant ropey collagen. B**, Foccally, in striking myxoid stroma, the cells are scarcely diffused, and the vascular pattern varies from plexiform to dilated intermediate-sized vessels. **C**, The multinucleate cells have hyperchromatic, pelmorphic and florte-like muclei. **D**, Scattered inflammatory cells, such as lymphocytes and plasma cells could be seen in myxoid stroma.

Grossly, the resected mass measured 5.2 × 3.8 × 3.6 cm with regular shape, and was well circumscribed, the cut surface showed grey-white in colour. Histologically, the tumor was made up of hypocelluar bland spindle cells admixed with scattered multinucleated cells and irregular ropey collagen in a diffuse myxoid stroma. There was no area of mature adipose tissue in the whole section. The spindle cells were uniform with hyperchromatic nuclei and inconspicuous nucleoli. The multinucleated cells had hyperchromatic nuclei arranged into a “floret-like” pattern, and showed marked nuclear atypia. However, the mitoses were rare. The vascular pattern mainly consisted of a few small or intermediate-sized, thick-walled vessels, focally, there was plexiform vascular pattern resembling that in myxoid liposarcoma. Moreover, Coarse “ropelike” collagen bands were randomly diffused in the cellular elements (Figure 1).

**Figure 2:**
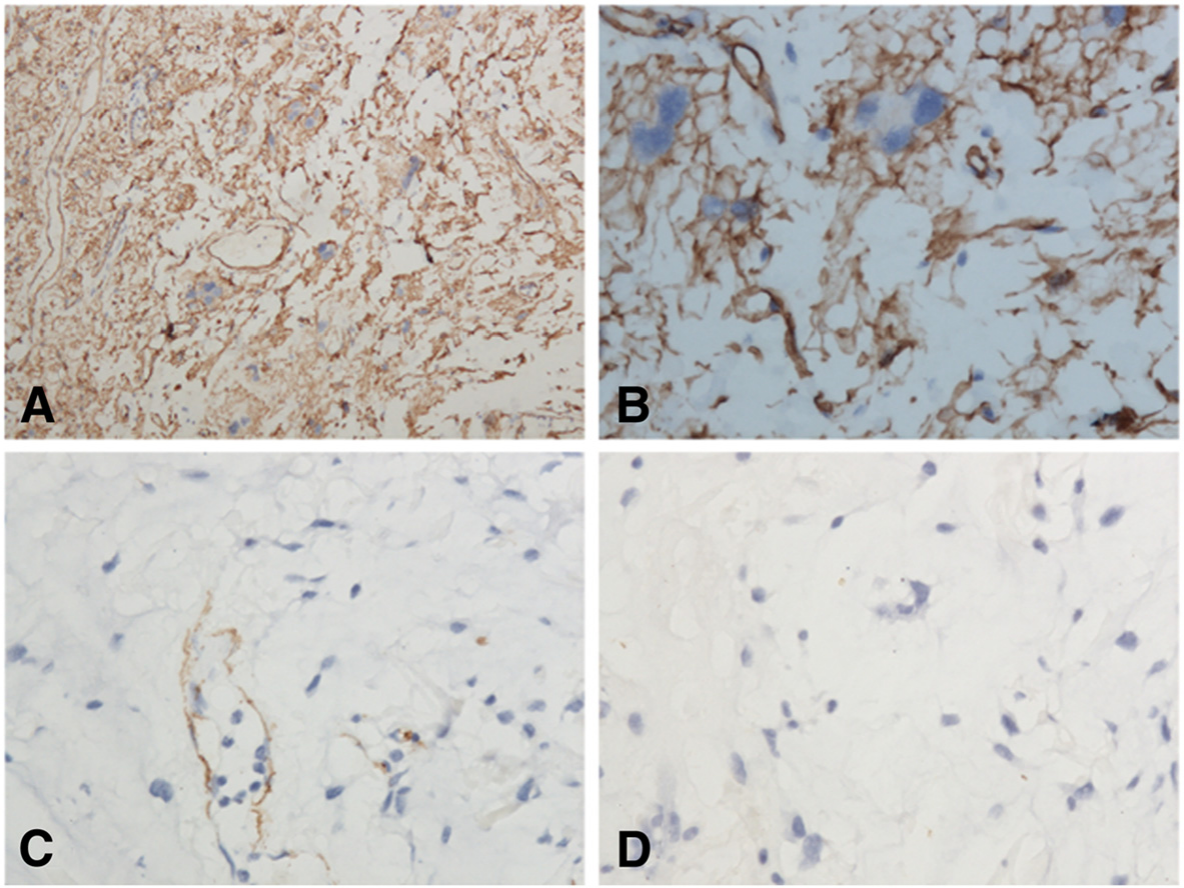
**A,Diffuse and strong CD34 staining highlighted the tumor cells. B**, The floret-like cells were positive for CD34. **C**, The spindle cells and floret-like cells were negative for SMA, while SMA staining highlighted the dilated vessel. **D**, All tumor cells including floret-like cells were negative for S-100.

Immunostaining showed that the tumor was positive for the Vimentin, Bcl-2 and CD34, and was negative for S-100, desmin, CD68 and α–SMA. Ki67 was expressed in less than 2% of all tumor cells (Figure 2). According to the morphological and immunohistochemical findings, the tumor was diagnosed as a pelomprphic lipoma.

## Discussion

Pleomorphic lipoma, considered as a variant of spindle cell lipoma, typically presents in older men with a median age of more than 55 year. The majority of the lesions occurs in the subcutis of the posterior neck, back, and shoulder area [1]. Less frequently, it can occur in palm [9], tonsillar fossa [10], orbit [11], tongue [12], vulva [13] and oral cavity [14]. Our reported case occurred in axilla, which was also a relatively uncommon location. Pleomorphic lipomas are benign tumors, can be readily treated by excision, although occasionally recurrences can happen.

Histologically, pelomorphic lipoma is largely composed of mature fat and bland spindle mesenchymal cells. In addition, multinucleated giant cells are scattered amid the spindle cells, and their nuclei are radically arranged in a “floret-like” pattern. The spectrum of histology shows a wide variation, and varies from tumor that resembles ordinary lipoma with few spindle cells to tumor that mainly consists of spindle cells with just a few fat cells. Cytologically, spindle cells have single elongated hyperchromatic nuclei and inconspicuous nucleoli, whereas multinucleated giant cells have irregular, hyperchromatic and significantly atypical nuclei. The mitoses of the two cell types are rare. “Ropelike” collagen bands are randomly distributed amid the cellular elements, usually an important diagnosis clue to pelomorphic lipoma. Some lesions may have extensive myxoid stroma, which can be a dominant feature and pose a diagnostic challenge. Some inflammatory cells including mast cells, lymphocytes and plasma cells are often scattered among the spindle cells. The vascular pattern usually consists of a few small or intermediate-sized, thick-walled vessels. In contrast, some examples have a prominent plexiform vascular pattern reminiscent of myxoid liposarcoma [15]. A pseudoangiomatous variant of pleomorphic lipoma has also been described [16]. Immunohistochemically, the spindle cells and “floret-like” cells are strongly positive for CD34, but negative for S-100 protein and smooth muscle actin [4,5]. Some cases can show desmin positive expression, which may lead to a misdiagnosis of a smooth muscle tumor [17].

The differential diagnosis of classic pleomorphic lipoma includes atypical lipomatous tumor/well-differentiated liposarcoma and pleomorphic liposarcoma. The typical pleomorphic lipoma usually arises in the subcutaneous tissue of shoulder or head and neck region. But, atypical lipomatous tumor/well-differentiated liposarcoma and pleomorphic lipoma usually arises in deep soft tissue of extremities or retroperitoneum. “Floret-like” giant cells in pleomorphic lipoma occasionally can also be seen in atypical lipomatous tumor/well-differentiated liposarcoma, which is not suitable for distinguishing them. The ropey collagen can not be seen in atypical lipomatous tumor/well-differentiated liposarcoma and pleomorphic liposarcoma, so it is useful for differential diagnosis. Moreover, pleomorphic lipoma lacks lipoblasts, which can be seen in the above two lesions [3].

However, if a tumor with the background of pleomorphic lipoma abosolutely lacks the fat cells, whether the tumor still should be diagnosed as a pleomorphic lipoma? In fact, it is debated by some pathologists. In our reported case, spindle cells and “floret-like” cells are scattered in the extensive myxoid stroma and the adipose tissue was absolutely absent. It poses a great diagnostic challenge. So far, there is only one literature that described two cases of pleomorphic lipoma with devoid of mature fat tissue. That is, Sachdeva MP et al. reported a review of 38 pleomorphic lipomas seen in consultation revealed 2 cases in which fat was absent. It was called the fat-free variant of pleomorphic lipoma [18]. They believed that diagnosis of pleomorphic lipoma mainly depended on the context of the tumor and nonlipomatous components rather than lipomatous components. In our case, because of the absence of fat, we first thought that it might be a myxoid fibrosarcoma. Myxoid fibrosarcoma is more common than pleomorphic lipoma in elderly male. The majority of them occur in extremities, rarely on the trunk or head and neck area. Histologically, myxoid fibrosarcoma usually consists of spindle cells and multinucleate giant cells, and is characterized by prominent elongated, curvilinear, thin-walled blood vessels. The spectrum of myxoid fibrosarcoma is also variable. However, myxoid fibrosarcoma tends to have more cellular atypia and mitosis activity. Moreover, myxoid fibrosarcoma usually lacks strong CD34 expression [19]. So, myxoid fibrosarcoma can be ruled out.

In addition, the differential diagnosis also includes giant cell fibroblastoma, pleomorphic fibroma and giant cell angiofibroma, which also show CD34 positive expression and possess multinucleated giant cells. Giant cell fibroblastoma is a juvenile form of dermatofibrosarcoma protuberans (DFSP). It affects predominantly infants and children. Its characterized feature is that multinucleated tumor cells often line slitlike pseudovascular spaces [20]. Pleomorphic fibroma is characterized by hypocellular dermal proliferations of spindle and irregularly shaped stellate or multinucleate cells with storiform and clefted hyalinized collagen [21]. Giant cell angiofibroma is a giant-cell-rich form of solitary fibrous tumor. It displays all the features of a classic SFT but is identified by pseudovascular spaces lined by multinucleated stromal giant cell [22]. Occasionally, multinucleated giant cells could also be seen in neurofibroma [23,24], which show CD34 and S-100 positive expression. Although our case lacks mature fat tissue, according to the morphological and immunohistochemical findings, at last, the tumor was diagnosed as a pelomprphic lipoma.

## Conclusion

Because of the relative rarity, variable histological spectrum and cellular pleomorphism, pleomorphic lipoma is misdiagnosed easily, especially when one unfamiliar with this entity. Pleomorphic lipoma lacking mature fat tissue is rarer, and may cause great diagnostic confusion. Because of the absence of adipocytes, it is usually thought as a nonlipomatous lesion rather than lipoma. To avoid the misdiagnosis, careful attention to histological features including spindle cells, “floret-like” cells, especially ropey collagen is essential for the correct diagnosis of pleomorphic lipoma.

## Consent

Written informed consent was obtained from the patient for publication of this case report and accompanying images. A copy of the written consent is available for review by the Editor-in Chief of this Journal.

## Competing interests

The authors declare that they have no competing interests.

## Authors’ contributions

LXY and WY participated in the histopathological evaluation, performed the literature review, acquired photomicrographs and drafted the manuscript. LY, SY and MY carried out the immunohistochemical stains evaluation. ZY and YJH conceived and designed the study. WEH gave the final histopathological diagnosis and revised the manuscript. All the authors read and approved the final manuscript.
